# Automatic Identification of Mobile and Rigid Substructures in Molecular Dynamics Simulations and Fractional Structural Fluctuation Analysis

**DOI:** 10.1371/journal.pone.0119264

**Published:** 2015-03-27

**Authors:** Leandro Martínez

**Affiliations:** Institute of Chemistry, University of Campinas—UNICAMP, Campinas, SP, Brazil; MRC National Institute for Medical Research, UNITED KINGDOM

## Abstract

The analysis of structural mobility in molecular dynamics plays a key role in data interpretation, particularly in the simulation of biomolecules. The most common mobility measures computed from simulations are the Root Mean Square Deviation (RMSD) and Root Mean Square Fluctuations (RMSF) of the structures. These are computed after the alignment of atomic coordinates in each trajectory step to a reference structure. This rigid-body alignment is not robust, in the sense that if a small portion of the structure is highly mobile, the RMSD and RMSF increase for all atoms, resulting possibly in poor quantification of the structural fluctuations and, often, to overlooking important fluctuations associated to biological function. The motivation of this work is to provide a robust measure of structural mobility that is practical, and easy to interpret. We propose a Low-Order-Value-Optimization (LOVO) strategy for the robust alignment of the least mobile substructures in a simulation. These substructures are automatically identified by the method. The algorithm consists of the iterative superposition of the fraction of structure displaying the smallest displacements. Therefore, the least mobile substructures are identified, providing a clearer picture of the overall structural fluctuations. Examples are given to illustrate the interpretative advantages of this strategy. The software for performing the alignments was named MDLovoFit and it is available as free-software at: http://leandro.iqm.unicamp.br/mdlovofit

## Introduction

Molecular Dynamics (MD) simulations are used to the study of the motions of macromolecular systems of high complexity, among which biomolecules are of utmost interest [1]. An important part of the analysis consists in the description of the structural fluctuations of the macromolecule [2, 3], which can be complex and hard to interpret from a functional perspective.

The two most common measures of structural fluctuations are the Root-Mean-Square-Deviation (*RMSD*) and the Root-Mean-Square-Fluctuations (*RMSF*) [4]. The *RMSD* is the average displacement of the atoms at an instant of the simulation relative to a reference structure, usually the first frame of the simulation or the crystallographic structure. The *RMSF* is a measure of the displacement of a particular atom, or group of atoms, relative to the reference structure, averaged over the number of atoms. The *RMSD* is useful for the analysis of time-dependent motions of the structure. It is frequently used to discern whether a structure is stable in the time-scale of the simulations or if it is diverging from the initial coordinates. Most times, the divergence from the initial coordinates is interpreted as a sign that the simulation is not equilibrated. When a simulation is equilibrated, that is, when the structure of interest fluctuates around a stable average conformation, it makes sense to compute the fluctuations of each subset of the structure (each atom, for example) relative to the average structure of the simulation, the *RMSF*.

However, these measures of conformational mobility are dependent on the strategy used for structural superposition. For proteins, the usual *RMSD* or *RMSF* computations involve the rigid-body alignment of the structures in each frame of the simulation to reference coordinates. The rigid-body alignment is very sensitive to the existence of subsets of the structure with high conformational fluctuations. High *RMSD*s or *RMSF*s might indicate the whole structure fluctuates, or might reflect only large displacements of a small structural subset within an overall rigid structure. As the structures studied by MD simulations become larger, it becomes increasingly common to find high *RMSD*s related to the large fluctuations of structural subsets that do not reflect the structural fluctuations of the macromolecule as a whole. Therefore, alignment methods that help to discriminate flexible from rigid structural subsets will be increasingly important for the analysis of MD simulations.

The limitations of the *RMSD* as a measure of structural variability are thoroughly studied in the context of protein structural alignment [5–7]. For the alignment of two structures to be robust, new alignment scores were defined such that the atoms that are the least displaced contribute with greater weigh. Thus, large differences in subsets of the structures being compared do not dominate the alignment. These scores are good and popular alternatives to *RMSD* minimization [6], except for two reasons: First, the optimization of these scores cannot be performed with the standard rigid-body alignment algorithm, which provides the global minimization of the *RMSD* [7, 8]; second, the interpretation of the structural fluctuations on the basis of the scores is not as direct and intuitive as that based on the mean displacement of atoms.

Improved alignment strategies can have complementary properties to other mobility analysis methods, such as Principal Component Analysis (PCA) [9]. If in a given simulation the largest structural fluctuations coincide with low frequency modes, the representation of these modes will be consistent with the structural representation of the alignment obtained. If not, the low frequency modes will not coincide with the largest fluctuations, and both images will be complementary. This might be important, as it has been already observed that slow modes obtained by PCA can be exclusive of a time window in the simulation [9].

We have previously shown that the minimization of structural alignment scores can be performed under the scope of Low-Order-Value-Optimization (LOVO) theory [7, 10]. In LOVO problems, the goal is to minimize an objective function that assumes the minimum value of a set of concurrent functions in the same domain [11]. Many problems, particularly the identification of outliers in linear and non-linear fitting, can be interpreted under LOVO theory. Interestingly, although the objective functions are generally non-smooth, optimization methods using derivatives can be used safely and efficiently [10, 11].

**Fig 1:**
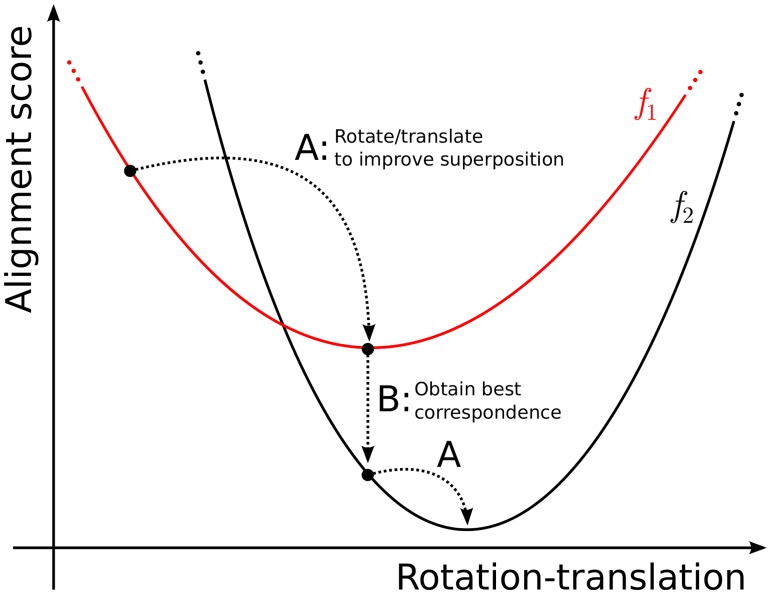
Sketch of the Low-Order-Value-Optimization (LOVO) problem in structural alignment: The goal is to minimize a function which assumes the lowest value of a set {*f*_*i*_} of concurrent functions in the same domain. For each correspondence between atoms, there exists a function *f*_*i*_ that provides the quality measure of the alignment (the score) as function of rotations and translations of one of the structures. The minimization of the score is performed by iterating between minimizing the *f*_*i*_ corresponding to the current correspondence between atoms (steps of type A), followed by obtaining the correspondence that minimizes the score for each rotation-translation (steps of type B). This procedure is guaranteed to converge with the appropriate choices of the methods used in steps A and B [7, 10, 11].

The interpretation of structural alignment within LOVO theory is sketched in Fig. 1. There are two challenges in the structural alignment problem: First, the determination of the correspondence between atoms of the two structures. Second, given the correspondence between atoms, one must determine the relative displacement that maximizes the quality of the superposition. Therefore, one can interpret the structural alignment as follows (Fig. 1): There is a set of functions {*f*_*i*_}, *i* being the index of each possible correspondence between atoms of the two structures. Each *f*_*i*_ assumes a value corresponding to the quality of the alignment (the score) as a function of the relative rotations and translations of one of the structures. The objective function, *F*, assumes the optimal value of the score for each relative displacement, that is, *F* = *min*(*f*_1_, …, *f*_*n*_) for each displacement. The goal of the alignment method is to minimize *F*. Therefore, as sketched in Fig. 1, the optimization of the superposition involves two types of iterative steps: A and B. Steps of type A consist of obtaining the best displacements of one the structures that optimize the score, for a given correspondence between atoms. Steps of type B consist of the determination of the correspondence that optimizes the score, for a given relative orientation of the two structures.

In general, protein alignment involves structures with different sequences and number of residues. Thus, it is necessary to determine which residues from one structure correspond to which residues from the other structure. This correspondence between atoms is usually obtained using Dynamic Programming [12]. Classic alignment methods, such as the *Structal* algorithm [5], use the rotations and translations provided by standard rigid-body superposition methods [8] to minimize the *RMSD* of the corresponding atoms. As we have shown [7], this can be improved by the replacement of the *RMSD* minimization step by the explicit maximization of the superposition score using smooth optimization methods.

In this paper, the minimization of the *RMSD* for fractions of the structure is shown to fit into the LOVO framework and to be useful for the analysis of protein flexibility in MD simulations.

Within MD simulations it is interesting that the alignment measure retains a direct interpretation in terms of structural fluctuations. Thus, the *RMSD* is still the preferred measure of structural similarity. Also, there is no ambiguity in the determination of the correspondence between atoms, since the alignment involves the same structure in different frames of the simulation. Given the correspondence, standard rigid-body alignment rotates and translates one of the structures minimizing the *RMSD*, thus obtaining an optimal alignment.

However, proteins can display distinct flexibilities in different structural elements. If a structural alignment is performed, for example for all C*α* atoms, the regions displaying greater displacements dominate the *RMSD*, and an incomplete picture of the mobility of the structure is obtained: Regions of high vs. low mobility might not be differentiated. Given a relative orientation of the two structures, the identification of the subsets with the lowest flexibilities consists in the determination of the list of pair of atoms with the smallest displacements. Thus, it consists in a step of type B of the general LOVO strategy represented in Fig. 1. If this subset is identified, one can obtain the minimal *RMSD* by performing a rigid-body superposition, consisting in a step of type A in Fig. 1. A new relative orientation of the two structures is thus obtained, and a new subset of atoms might be identified as the ones with the least displacements. The iterative definition of the subsets to be aligned and the actual rigid-body superposition leads to the optimal alignment of the subsets with the lowest flexibilities, without requiring previous knowledge of these subsets. The proper definition of this algorithm and its implementation constitutes the present contribution. A software is provided, and examples illustrating the interpretative advantages of this approach are shown. The computer program is named MDLovoFit, and is available as free software at: http://leandro.iqm.unicamp.br/mdlovofit.

## Materials and Methods

Let {**x**_*i*_(*t*)}, **x** ∈ *R* ^3^, be the coordinates of the *N* atoms of a structure, lets say a protein, at instant *t* of the simulation. Let $\left\{{\text{x}}_{i}^{ref}\right\}$ be the coordinates of a reference structure, for example the crystallographic model, or the structure at *t* = 0. The *RMSD* of structure at time *t* relative to the reference structure is (Equation 1)

$$\begin{array}{c} RMSD\left(t\right)=\sqrt{\frac{1}{N}\sum_{i}^{N}\left|\right|x_{i}\left(t\right)-x_{i}^{ref}{\left|\right|}^{2}}, \end{array}$$ (1)

where ∣∣⋅∣∣ is the euclidean norm. We define the mean square displacement, *MSD*, per-atom as

$$\begin{array}{c} MSD_{i}\left(t\right)=\left|\right|x_{i}\left(t\right)-x_{i}^{ref}{\left|\right|}^{2}, \end{array}$$ (2)

where {*MSD*_*i*_(*t*)} is a list of *N* per-atom deviations.

The alignment algorithms proceeds as follows:

1. Compute, using any relative orientation of the two structures, the set of per-atom deviations {*MSD*_*i*_(*t*)}. Usually, this set is computed after a standard rigid-body alignment of the two structures, but this is not conceptually necessary.
2. Sort the per-atom deviations {*MSD*_*i*_(*t*)} in increasing order.
3. Choose the fraction *ϕ* (*ϕ* < 1) of atoms displaying the smallest deviations, and perform a rigid body alignment for this substructure.
4. Translate and rotate the entire structure according to movements of the rigid-body alignment of step 3.
5. Sort {*MSD*_*i*_(*t*)} in increasing order. Compute the sum of the *N*_*L*_, *N*_*L*_ = *ϕN*, smallest *MSD*_*i*_(*t*), denoted by *MSD*_*L*_(*t*)
6. If *MSD*_*L*_(*t*) has converged, stop. Otherwise, go to step 3.

This algorithm is guaranteed to converge to local minimizers by LOVO theory [11] and by the arguments presented in Fig. 1. It converges quite rapidly in practical applications. In order to obtain global minimizers, multiple initial random choices of *N*_*L*_ atoms are used. The globalization of the method is important particularly for proteins with multiple domains, in which different subsets of similar mobility can be identified. Different initial guesses of the correspondence may lead to the identification of different domains of small flexibility.

The above algorithm was implemented for the analysis of molecular dynamics simulations trajectories. A software named MDLovoFit was developed and is available under general public licenses at

http://leandro.iqm.unicamp.br/mdlovofit.

Currently the software requires the trajectory to be in PDB format. A tutorial is provided such that mobility analysis similar to the ones presented in the Results and Discussion section can be performed by the user.

The alignment of 100 frames of a 200 residue structure using this method takes about 5 seconds in a typical personal computer. Mapping all possible *ϕ* values (with a 0.01 step) takes less than five minutes. Therefore, the computational time required by these methods is not an issue for their practical use. By default, 100 initial points are tested, but this parameter can be controlled by the user.

Once the method converges, we compute: 1) The total *RMSD* of the structure, that we call *RMSD*_*T*_(*t*). 2) The *RMSD* of the fraction of atoms that were automatically identified by the iterative procedure as the least displaced, *RMSD*_*L*_(*t*). 3) The *RMSD* of the atoms that were not explicitly considered in the alignment and therefore display high structural fluctuations, *RMSD*_*H*_(*t*).

In the current implementation, focused on the analysis of the structural fluctuation of proteins, the alignment is performed for C*α* atomic coordinates. Given the subset of atoms that are considered in each alignment, the classical rigid-body alignment algorithm of Kearsley [8] is used. We use FlashSort1 [13] for sorting {*RMSD*_*i*_(*t*)} in increasing order.

The fraction *ϕ* of atoms to be considered explicitly at each alignment is a choice of the investigator. If the size of a particular substructure is known, it can be defined directly. At the same time, the software provided also implements the scanning of the full range of *ϕ* values, and the output of *RMSD*_*L*_, *RMSD*_*H*_ and *RMSD*_*T*_ as a function of *ϕ*.

The software also outputs the *RMSD* per-atom and the atoms identified as the least mobile ones in the *b*-factor and occupancy fields of a trajectory PDB file which can be used for visualization, for example, with VMD [4]. Optionally, the *RMSF* of C*α* atoms is also computed.

## Results and Discussion

Two sets of simulations are presented to illustrate the advantages of the proposed alignment method.

First, we analyze the protein structural fluctuations of two simulations of the *Burkholderia cepacia* lipase (PDB id. 1YS1) [14] in mixtures of water and a co-solvent, sorbitol, that is known to improve the protein stability. The systems were built with Packmol [15] and simulations were performed with NAMD [1] at 298K for 40 ns, using standard simulation protocols similar to those described previously [16].

**Fig 2:**
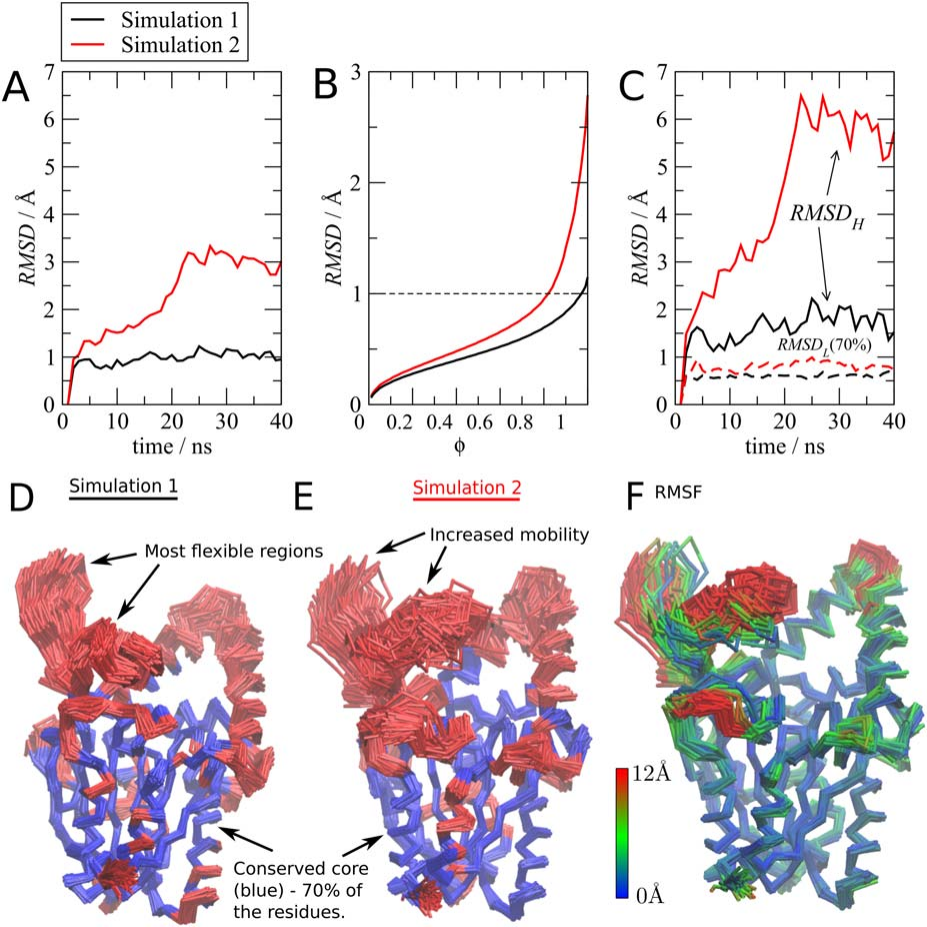
Analysis of the mobility of two simulations of a *Burkholderia cepacia* lipase [14] in mixtures of water and sorbitol. Different *RMSD* profiles are observed: (A) The standard *RMSD* of Simulation 1 (black) is much lower than the *RMSD* of Simulation 2 (red). (B) *RMSD* as a function of the fraction of the atoms considered in the alignment. These plots indicate that in Simulation 2 (red), there is a subset of about 25 to 30% of the atoms which are responsible for the greater overall *RMSD* observed in panel (A). (C) In both simulations, 70% of the atoms can be superposed to less than 1Å (*RMSD*_*L*_—dotted lines, black for Simulation 1, red for Simulation 2). The remaining 30% of the atoms behave differently in each simulation (*RMSD*_*H*_—solid lines, same colors). (D) and (E) Superposition of the frames and coloring of the 70% least mobile atoms (blue) and 30% most mobile atoms (red) provides the structural basis for the differential *RMSD*s. (F) Structures of Simulation 2 colored according to the *RMSF* of each residue relative to the initial structure after alignment. All these plots and figures can be obtained from the output of MDLovoFit.

The standard C*α* *RMSD*s of the lipase in the two simulations are represented in Fig. 2A, computed relative to the first frames of the simulations. In Simulation 1 (black lines), the C*α* *RMSD* is below 1Å for most of the simulation. The protein structure, therefore, is very stable, and does not diverge from the initial structure. In Simulation 2 (red lines) the standard *RMSD* (Fig. 2A) increases much more and becomes greater than 3Å at about 25 ns. Clearly, the structure undergoes some structural change.

Using MDLovoFit, we analyzed the fluctuations of the structure by performing structural alignments for subsets of the protein corresponding to different fractions *ϕ* of the total number of C*α* atoms, as shown in Fig. 2B. The algorithm automatically detects the subset of atoms with smallest *RMSD* for each *ϕ*. It is possible to identify, in both simulations, subsets of at least 70% of the atoms that can be superposed to the initial frames to less than 1Å. However, a sharp increase in *RMSD* is observed for Simulation 2, starting from *ϕ* = 0.70 (Fig. 2B). Therefore, there is in both simulations a well preserved core formed by about 70% of the structure. The divergence in the C*α* *RMSD*s is caused by the fluctuations of a subset of the atoms.

This is confirmed by the computation of the fluctuations of these subsets along the simulation. In Fig. 2C, MDLovoFit was used to compute the time-dependent structural deviations of the (automatically detected) subset of 70% of atoms with the smallest displacements. The deviations of the conserved core (*RMSD*_*L*_: dotted black and red lines in Fig. 2C) are persistently below 1Å, and do not display any sign of divergence from the initial structure. Therefore, in both simulations, the protein displays a preserved core. At the same time, the structural deviations of the remaining 30% of the structure are clearly different. While in Simulation 1 these atoms fluctuate stably around 1.5Å, in Simulation 2 these atoms diverge from the initial structure by more than 6Å (*RMSD*_*H*_).

Figs. 2D and E show the structural superposition of the trace of the protein along the simulations. The atoms are colored in blue if they were explicitly used in the alignment (that is, if they were identified as belonging to the 70% least displaced), and red otherwise. The PDB files used for producing these figures are also automatically provided by MDLovoFit. We note that the blue atoms in both images are similar, thus the conserved cores of the protein are the same. The most mobile regions of the structure are also similar, despite the fact that their displacements are different from one simulation to the other. A loop, displayed at the top left of the images, is responsible for the much greater structural deviations of Simulation 2 relative to Simulation 1. Fig. 2E illustrates the *RMSF* of each C*α* atom relative to the reference structure, at each frame. A quantitative picture of the displacements is thus obtained. The *RMSF* data per frame, used for producing this plot is also provided by the MDLovoFit package.

These results are biophysically relevant. The active site of the enzyme is located in a groove in the region with the lowest mobility, exposed to the solvent and close to the boundary between the upper blue and red regions in Figs. 2D and E. The regions displaying the lowest mobilities, in blue, provide support for the correct relative positioning of the active site residues. The mobile (red) regions might gate the entrance and release of substrate and products, as observed for other similar enzymes [17]. In this particular case, the simulations intend to study of the stabilization of the protein promoted by a co-solvent, and the method has shown that the core of the protein is preserved in independent simulations, in spite of the fact that the global RMSDs are different. This is a very common scenario in MD studies, where multiple simulations are performed, and for which it is important to recognize the structural features that are consistent within the simulation set, avoiding the overinterpretation of incidental fluctuations.

In summary, the use of fractional alignments allowed for the recognition that, in two simulations displaying different overall structural fluctuations, there exists a common preserved core. Furthermore, the subset of the structure that is responsible for the differential deviations is identified. Importantly, *RMSD* measures are attributed to the displacements of each subset, thus the quantitative interpretation of the scale of the displacements is straightforward.

**Fig 3:**
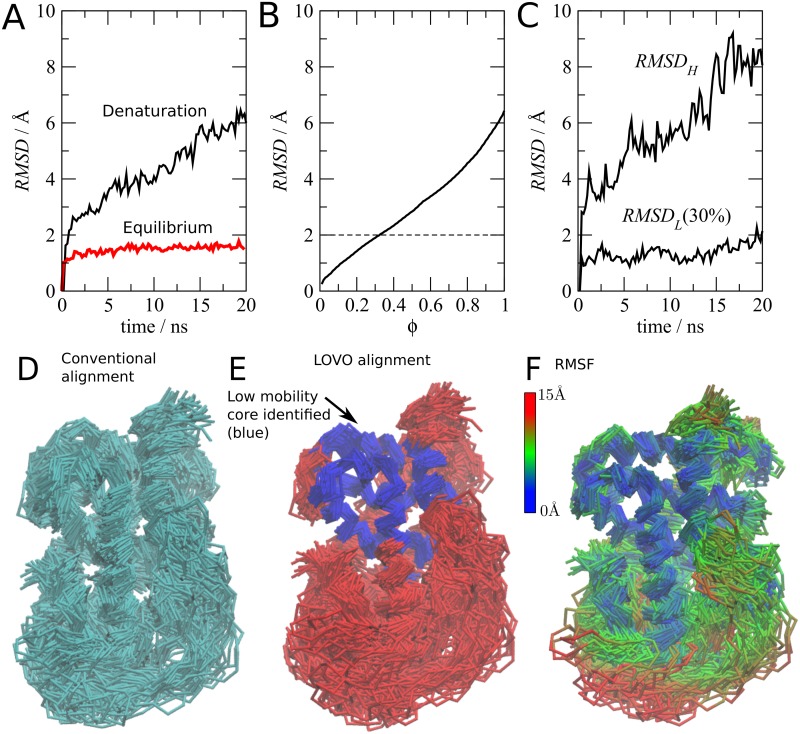
Analysis of the mobility of a simulation of the Ligand Binding Domain Thyroid Hormone Receptor-*β*, in which the structure diverges with time [19]. (A) The standard C*α* *RMSD* indicates an unfolding process in a high temperature simulation (black). The *RMSD* of an equilibrium room-temperature simulation is also shown (red). (B) The alignment between the first and last conformations of the unfolding simulation with variable *ϕ* indicates that there is a persistent subset of about 30% of backbone atoms that can be aligned to 2Å. (C) The 30% least mobile atoms display structural deviations of about 2Å relative to the initial structure (*RMSD*_*L*_), while the remaining structure diverges (*RMSD*_*H*_). (D) Superposition of the unfolding simulation frames using standard C*α* alignment. (E) Superposition using MDLovoFit for 30% of C*α* atoms (blue) highlights the existence of a well preserved structural core. The 70% most mobile atoms are shown in red. (F) Structure colored according to the *RMSF* of each residue relative to the initial structure after alignment.

The second example consists of a simulation of a structure that seemingly diverges in time. Fig. 3 shows the analysis of the mobility of the Ligand Binding Domain of the Human Thyroid Hormone Receptor-*β* (PDB id. 3JZC) [18] in a high-temperature (498K) simulation used for the study of its denaturation mechanisms. All details of these simulations were published previously [19].

As expected from a simulation in denaturing conditions, the structure progressively diverges from the first frame of the simulation, as shown by the C*α* *RMSD* in Fig. 3A (black). The RMSD of a typical equilibrium simulation, at 298K, is also shown (red). The maximum divergence of the structure from its initial coordinates, in equilibrium conditions, is smaller than 2Å in the time-scale of this simulation. We obtained with MDLovoFit the optimal *RMSD* of subsets of the structure with variable sizes, in denaturing conditions, by varying the fraction *ϕ* of atoms considered in the alignment. The *RMSD* obtained for each fraction *ϕ* is shown in Fig. 3B. Differently from the simulations of the previous example, only a small fraction of the atoms can be nicely superposed. However, the method detected that about 30% of the atoms can be aligned to an *RMSD* of about 2Å (Fig. 3B), that is similar to the fluctuations observed in the equilibrium simulation.

Therefore, the MDLovoFit alignment using 30% of the atoms throughout the simulation was performed. The mobility of this subset of atoms is then computed independently of the displacements of the remaining structure. Fig. 3C displays the deviations of the 30% least mobile atoms identified by the method (*RMSD*_*L*_) and of the remaining 70% C*α* atoms (*RMSD*_*H*_). The subset of the 30% least mobile atoms is consistently and stably aligned to less than 2Å up to almost 20 ns of simulation. The 70% atoms not used for the superposition diverge systematically from the initial structure. Therefore, we were able to identify that there is a subset of atoms which preserves the initial conformation, despite the denaturing conditions.

Fig. 3D displays the standard rigid-body alignment of the protein structure along the simulation. It is not easy to discriminate regions of higher or lower mobility from this superposition. Fig. 3E, on the other side, displays the alignment of the 30% least mobile atoms, which are colored in blue according to the MDLovoFit automatic classification. The rigid core of the protein is now apparent. The *RMSF* of each residue relative to the first frame, plotted in Fig. 3F, illustrates quantitatively the displacements.

Therefore, by using the MDLovoFit alignment methodology, we were able to perceive that there is a stable subset of the protein which resists denaturation in the time-scale of the simulations performed.

In this example, the results are also interesting from the biophysical point of view. In Fig. 3E, the lower part of the structure, which diverges structurally, contains the ligand binding pocket. As we reported previously [19], the ligand protects the structure from denaturation by linking the secondary structural elements that, in the example shown here without ligand, diverge the most from the crystallographic model.

## Conclusion

We propose an algorithm for structural alignment to be used in the interpretation of the mobility in MD simulations. The method allows the computation of the *RMSD* of mobile versus rigid subsets of the structure that are identified automatically. The interpretation in terms of the fluctuations of the structures is direct and intuitive, because the displacements are quantified by the *RMSD* of each subset. It is possible to identify the number of atoms of mobile and rigid subsets, and we provide tools for the visualization of the results on the protein structure. The analysis of the structural fluctuations with the present method provides a more complete picture of structural fluctuations in MD simulations, avoiding the incorrect or incomplete interpretation of the overall fluctuations. We have applied these methods in previous MD studies [16, 20], so we hope they can be useful to other researchers.
